# Valproate Alters Dopamine Signaling in Association with Induction of Par-4 Protein Expression

**DOI:** 10.1371/journal.pone.0045618

**Published:** 2012-09-24

**Authors:** Saebom Lee, Jaehoon Jeong, Young-Un Park, Yongdo Kwak, Seol Ae Lee, Haeryun Lee, Hyeon Son, Sang Ki Park

**Affiliations:** 1 Division of Molecular and Life Science, Department of Life Science, Biotechnology Research Center, Pohang University of Science and Technology, Pohang, Korea; 2 Division of Integrative Biosciences and Biotechnology, Pohang University of Science and Technology, Pohang, Korea; 3 Departments of Biochemistry and Molecular Biology and Physiology, College of Medicine, Hanyang University, Seoul, Korea; Chiba University Center for Forensic Mental Health, Japan

## Abstract

Chromatin remodeling through histone modifications has emerged as a key mechanism in the pathophysiology of psychiatric disorders. Valproate (VPA), a first-line medication for bipolar disorder, is known to have histone deacetylase (HDAC) inhibitor activity, but the relationship between its efficacy as a mood stabilizer and HDAC inhibitory activity is unclear. Here we provide evidence that prostate apoptosis response-4 (Par-4), an intracellular binding partner of dopamine D2 receptors (DRD2), plays a role in mediating the effectiveness of VPA. We found that chronic VPA treatment enhanced the expression of Par-4 in cultured neurons and adult mouse brains. This Par-4 induction phenomenon occurred at the transcriptional level and was correlated with an increase in histone H3 and H4 acetylation of the Par-4 promoter regions. Furthermore, chronic VPA treatment potentiated the suppression of the cAMP signaling cascade upon dopamine stimulation, which was blocked by sulpiride treatment. These results indicate that VPA potentiates DRD2 activity by enhancing Par-4 expression via a chromatin remodeling mechanism.

## Introduction

Bipolar disorder, also known as manic depression, is a severe mental illness affecting about 1% of the population and has multifaceted potential causes [1]. The common symptoms of bipolar disorder are serious shifts in mood, which include ‘cyclic mood swings’ characterized by rapid emotional changes from manic to depressive episodes [2]. Extensive studies have generated a variety of hypotheses for the molecular mechanisms underlying bipolar disorder, however, the definite pathogenic mechanism has yet to be defined.

One of the most intriguing recent developments in the field is the emergence of chromatin remodeling events associated with the pathophysiology of psychiatric disorders [3], [4], [5], [6]. The nucleosome, which is the fundamental unit of chromatin, allows DNA and histone complexes to form open or closed states of transcription units based on the modification of histone tails, including acetylation [7]. The acetylation states of histone octamers are determined by the relative activities of histone acetyl transferases (HATs) and histone deacetylases (HDACs), which correlate with the expression level of target genes [8]. This remodeling of the chromatin structure in the promoter regions of genes related to mood control appears to be important for the efficacy of various antidepressants and mood stabilizers [6]. In this regard, HDAC inhibitors have gained significant attention for their potential use for the treatment of mood disorders [5], [9].

Valproate (VPA) is a short-chain fatty acid with HDAC inhibitor activity that is widely prescribed as one of the first-line medications for epilepsy and bipolar disorders [10], [11], [12], [13]. Chronic VPA treatment in bipolar patients often restricts the frequency of mood swings to either the manic or depressive state [14]. As VPA is known to inhibit HDAC1 and presumably other HDACs, it has been proposed that its effects may be mediated in part by histone modifications of specific target gene loci that are functionally associated with mood stabilization [15].

In this study, we provide evidence that VPA engages in dopamine signaling pathways through the induction of Par-4, an intracellular modulator of DRD2 activity, by mediating chromatin remodeling of the Par-4 promoter region [16]. The results presented here may provide a novel mechanistic link between chromatin remodeling and dopamine signaling in the mood stabilization mediated by VPA.

## Results

### Par-4 expression is enhanced by chronic VPA treatment

**Figure 1:**
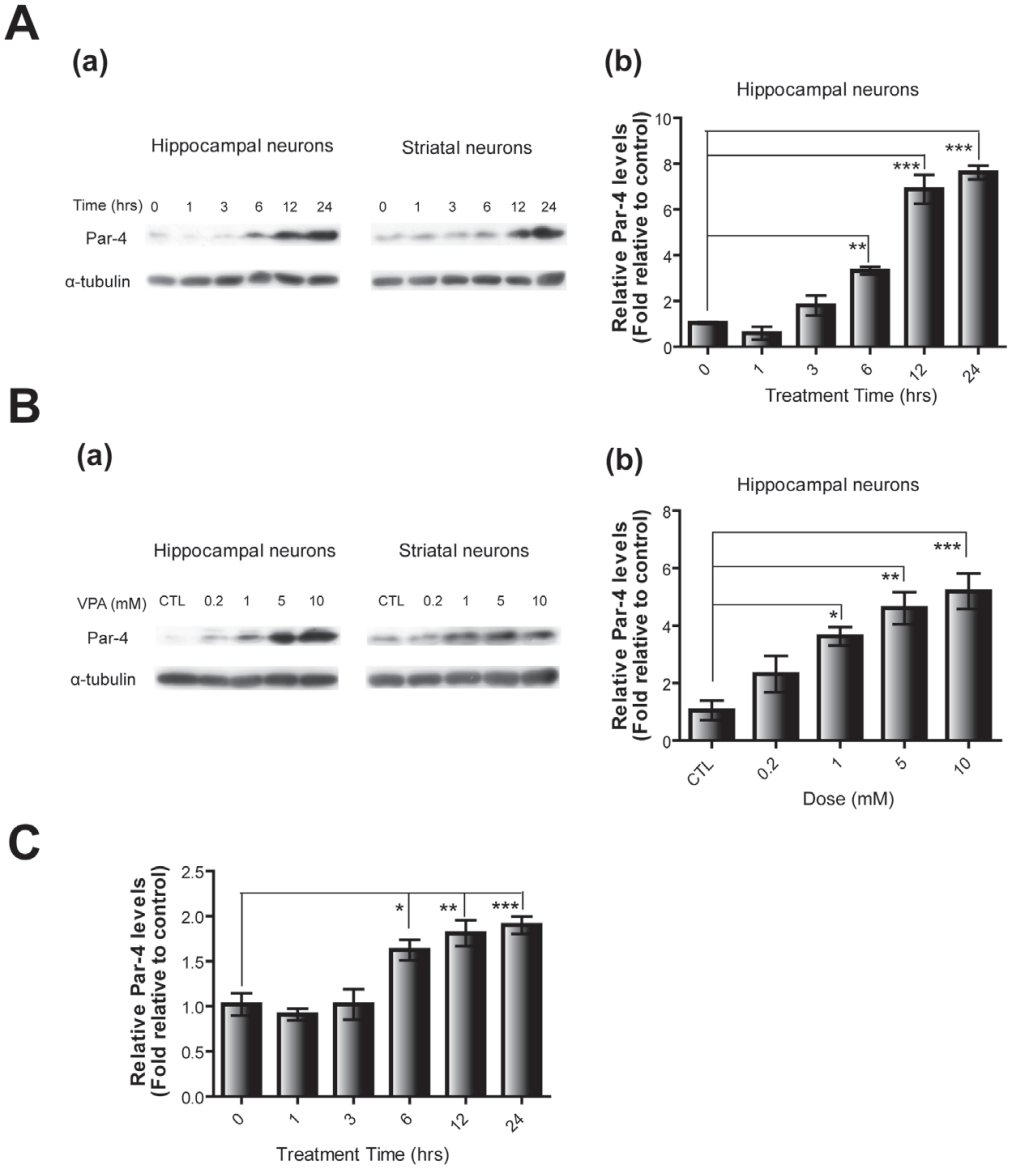
Induction of Par-4 expression by chronic VPA treatment. Time-dependent induction of Par-4 protein in primary cultured neurons. Cultured hippocampal and striatal neurons (DIV 7) were treated with 1 mM VPA. Par-4 protein levels relative to α-tubulin were analyzed by western blot (a) and subject to densitometric analysis (b). Error bars are mean ± SEM values (n = 4; *p<0.05, **p<0.01, ***p<0.001; One-way ANOVA with Bonferroni post hoc test). A. Dose-dependent increase of Par-4 proteins in mouse primary cultured neurons. Cultured primary neurons (DIV 7) were incubated with VPA at doses indicated for 24 hrs, and the Par-4 expression levels were analyzed as in (a). Par-4 levels relative to α-tubulin were analyzed (b). Error bars are mean ± SEM (n = 4; *p<0.05, **p<0.01, ***p<0.001; One-way ANOVA with Bonferroni post hoc test). B. Increased Par-4 mRNA after VPA treatment. Cultured hippocampal neurons (DIV 7) were treated with 1 mM VPA. Fold inductions of Par-4 mRNA relative to GAPDH were assessed by quantitative real-time PCR. Error bars represent mean ± SEM (n = 4; *p<0.05, **p<0.01, ***p<0.001; One-way ANOVA with Bonferroni post hoc test).

Par-4 has been proposed as a positive modulator of intracellular DRD2 signaling, which is thought to be associated with normal mood-associated behavior in experimental animals [16]. Because VPA is a first-line mood stabilizer, we examined a potential link between Par-4 and VPA efficacy. Interestingly, when cultured mouse primary neurons at DIV 7 were exposed to VPA, Par-4 protein levels increased in a treatment time- and concentration-dependent manner (Fig. 1A and B). A remarkable increase was observed after 6 hrs of treatment, indicating that significant Par-4 induction requires prolonged VPA treatment. To assess the induction of Par-4 at the transcription level, primary cultured hippocampal neurons at DIV 7 were treated with VPA for different durations, and Par-4 mRNA levels were examined using quantitative real-time PCR (Fig. 1C). A prominent increase in Par-4 mRNA was detected after 6 hrs of VPA treatment and correlated with the time of protein induction (Fig. 1C), indicating that increases in Par-4 gene transcription are responsible for the elevated Par-4 protein observed upon VPA treatment.

### Par-4 induction by chronic VPA treatment involves HDAC inhibition

**Figure 2:**
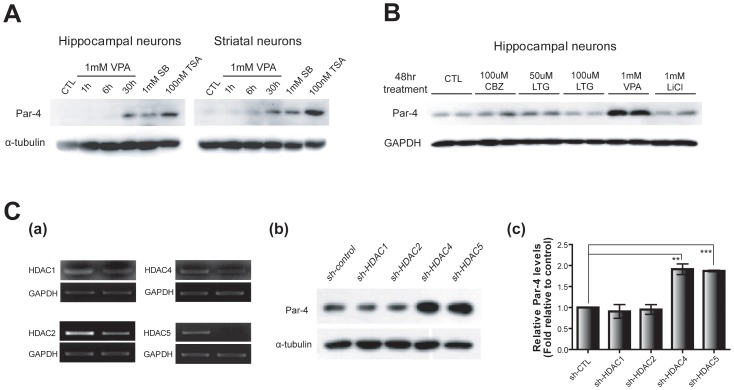
Involvement of HDACs in the induction of Par-4. A. Increased Par-4 expression upon chronic treatment of HDAC inhibitors in primary cultured neurons. Cultured neurons (DIV 7) were treated with 1 mM VPA. Sodium butyrate (SB, 1 mM) and trichostatin A (TSA, 100 nM) were treated for 30 hrs, and Par-4 protein levels were analyzed by western blot. B. Effect of common mood stabilizers. Carbamazepine, lamotrigine, and lithium chloride were tested in addition to VPA. Hippocampal neurons (DIV 7) were incubated with the indicated drugs for 48 hrs, and Par-4 protein levels were analyzed by western blot. CBZ, carbamazepine and LTG, lamotrigine. C. Involvement of HDAC4 and HDAC5 in Par-4 induction. Knock-down efficiency of shRNA constructs targeting HDACs was measured by semi-quantitative RT-PCR (a). Increase in Par-4 protein after HDAC knock-down. CAD cells were transfected with shRNA constructs followed by 5 days of differentiation. Cell lysates were analyzed by western blot to detect changes in Par-4 protein levels after knock-down of specific HDACs (b). Fold changes of Par-4 protein levels relative to the control sample (c). Error bars represent mean ± SEM. (n = 3; *p<0.05, **p<0.01, ***p<0.001; Student's t-test).

Because VPA is known to mediate HDAC inhibition, we tested whether the transcriptional induction of Par-4 is caused by changes in HDAC activities. To accomplish this, we first examined if the Par-4 gene is affected by known HDAC inhibitors, such as sodium butyrate (SB) and trichostatin A (TSA). Cultured hippocampal and striatal neurons (DIV 7) were incubated with 1 mM SB and 100 nM TSA for 30 hrs, and Par-4 protein levels were examined. Chronic treatment with HDAC inhibitors effectively increased the Par-4 protein levels, indicating that the Par-4 gene is sensitive to HDAC inhibition. We also tested if other mood stabilizers including carbamazepine (CBZ), lamotrigine (LTG), and LiCl have a similar effect on Par-4 induction. After 48 hrs of treatment, CBZ, LTG, and LiCl did not significantly increase Par-4 protein levels in cultured hippocampal neurons (Fig. 2B), indicating the relative specificity of VPA for Par-4 induction. Collectively, these results support the idea that transcriptional upregulation mediated by chromatin remodeling may be involved in Par-4 induction by chronic VPA treatment.

We next attempted to scrutinize which HDACs are primarily involved in chronic VPA-mediated upregulation of Par-4. For this, we generated shRNA constructs for specific HDACs and confirmed effective knock-down of HDACs using semi-quantitative RT-PCR in differentiated CAD cells, which showed robust VPA-dependent Par-4 induction (Fig. 2C-a and data not shown). After 5 days of differentiation with simultaneous knock-down of HDACs, we observed a consistent induction of Par-4 by the knock-down of HDAC4 and HDAC5 (Fig. 2C-b and c), suggesting that HDAC4 and HDAC5 may mediate transcriptional activation of the Par-4 promoter region upon chronic VPA treatment.

### VPA induces chromatin remodeling at the Par-4 promoter region

**Figure 3:**
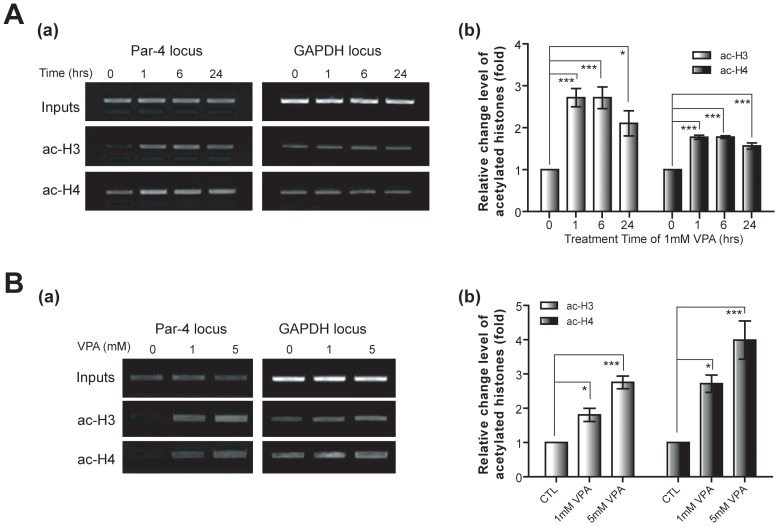
VPA-induced histone modifications at the Par-4 promoter region. A. Increased acetylation of histone H3 and H4 at the Par-4 promoter region in a time-dependent manner. Hippocampal neurons (DIV 7) were incubated with 1 mM VPA for 24 hrs and subjected to chromatin immunoprecipitation (ChIP) assay (a). Fold changes in ac-H3 and ac-H4 at the Par-4 promoter regions are shown in (b) relative to control. Error bars are mean ± SEM (n = 4; *p<0.05, **p<0.01, ***p<0.001; One-way ANOVA with Bonferroni post hoc test). B. Dose-dependent increases in ac-H3 and ac-H4 after chronic VPA treatment. Hippocampal neurons (DIV 7) were treated with increasing concentrations of VPA for 24 hrs and subject to ChIP assay (a). Relative fold changes in ac-H3 and ac-H4 at the Par-4 promoter regions were normalized to GAPDH levels (b). Error bars are mean ± SEM (n = 4; *p<0.05, **p<0.01, ***p<0.001; One-way ANOVA with Bonferroni post hoc test).

To further investigate the link between VPA and HDAC inhibition, we measured the levels of acetylated histones H3 (ac-H3) and H4 (ac-H4) in the Par-4 promoter region using cultured hippocampal neurons at DIV 7. Using chromatin immunoprecipitation (ChIP) assays with anti-ac-H3 and ac-H4 antibodies, a significant increase in ac-H3 and ac-H4 levels were observed at the Par-4 promoter region after 24 hrs of VPA treatment (Fig. 3A and B). The observed increases in ac-H3 and ac-H4 levels were also time- and dose-dependent, suggesting that transcriptional activation of Par-4 by VPA is mediated by increased H3 and H4 acetylation in the Par-4 promoter region.

### Chromatin remodeling at Par-4 promoter region in the brain

**Figure 4:**
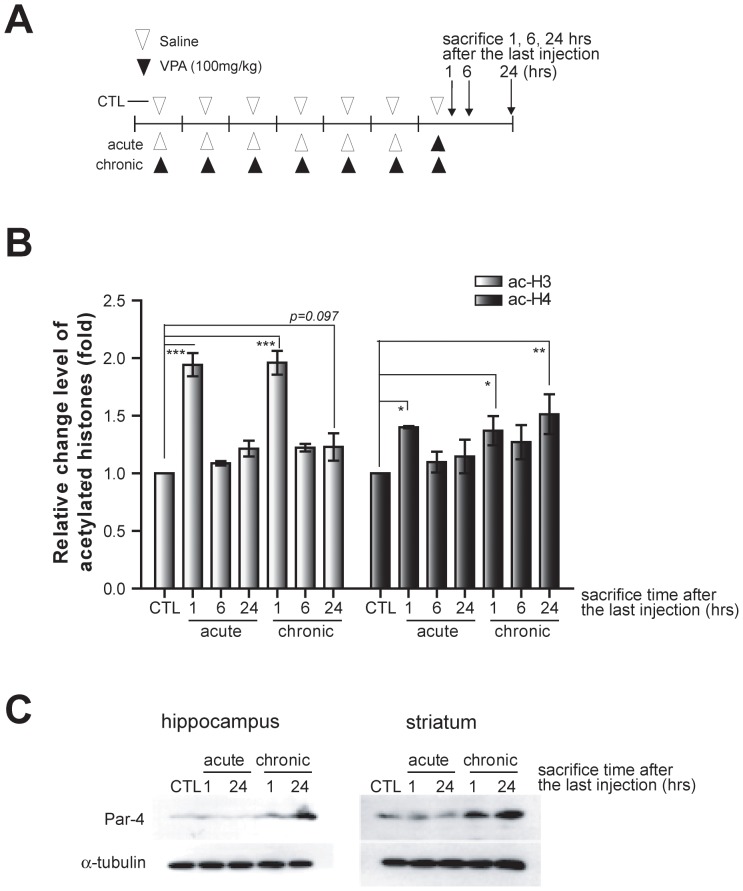
Dynamic histone modifications at the Par-4 promoter region in the brain. A. Experimental scheme of daily intraperitoneal VPA injection. ChIP assays were performed using hippocampal lysates. The acute treatment group was injected with saline for 6 days and VPA on the last day. Mice were sacrificed at the indicated time points after the last injection. The chronic treatment group was injected with 100 mg/kg of VPA for 7 days and sacrificed at the indicated time points after the last injection. B. Dynamic changes in histone modifications at the Par-4 promoter region in the brain. Mice (C57BL/6, male, 9 weeks of age) were intraperitoneally injected with saline or VPA (100 mg/kg per day) as indicated in (A). Ac-H3 and ac-H4 levels were normalized to GAPDH, and relative fold changes are shown compared to the CTL group. Error bars are mean ± SEM (n = 3; *p<0.05, **p<0.01, ***p<0.001; One-way ANOVA with Bonferroni post hoc test). C. Induction of Par-4 protein levels by chronic VPA treatment in the adult mouse brain. Par-4 protein levels were measured by western blot from hippocampal and striatal extracts.

To assess whether these histone modifications occur in physiological conditions, adult mice (C57BL/6, 9 weeks, male) were injected with 100 mg/kg VPA intraperitoneally, as shown in Figure 4A. Experimental groups were divided into three groups, including control (CTL; saline for 7 days), acute (saline for 6 days+VPA on the last day), and chronic (VPA for 7 days). Mice were sacrificed at the indicated time points after the final injection, and hippocampal regions were isolated for ChIP assays using anti-ac-H3 and ac-H4 antibodies. A significant increase in ac-H4 and a less robust effect in ac-H3 levels were observed after chronic VPA treatment, which correlated with the induction of Par-4 protein (Fig. 4B). However, these results in the brain were not as robust as those observed in cultured neurons, which may reflect the more complex experimental condition, for example, the rapid rate of VPA clearance in the brain [17]. Interestingly, an increase in ac-H3 and ac-H4 was shown by ChIP assay after acute VPA treatment, whereas the protein expression at those time points was largely unaffected (Fig. 4C), which may reflect more complex intermediary processes between chromatin remodeling and actual enhancement of transcription. Taken together, our data support the notion that the Par-4 gene is upregulated by changes in the chromatin state of Par-4 promoter region mediated by chronic VPA treatment in the brain.

### Chronic VPA treatment alters DRD2 signaling

**Figure 5:**
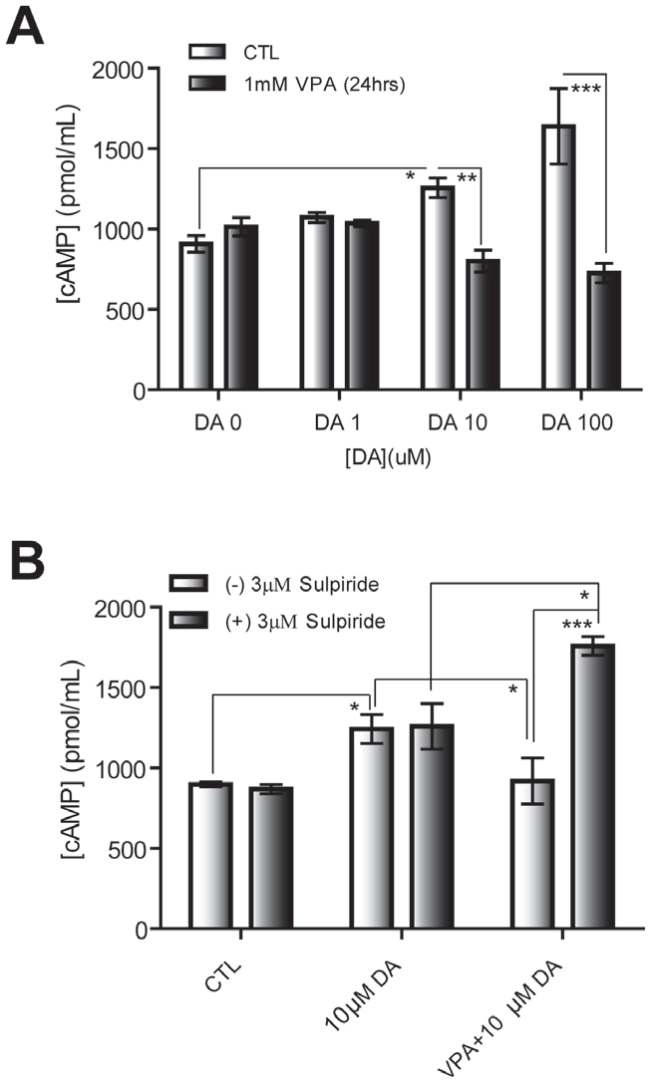
Activation of DRD2 signaling by chronic VPA treatment. A. Altered cAMP-response profile by VPA treatment in cultured striatal neurons. Cultured striatal neurons (DIV 7) were treated with increasing concentrations of dopamine (DA) for 30 min in the presence or absence of 1 mM VPA (24 hrs). cAMP levels in the cell lysates were measured using cAMP enzyme-immunoassays. Error bars are mean ± SEM. (n = 4, *p<0.05, **p<0.01, ***p<0.001; One-way ANOVA with Bonferroni post hoc test). B. VPA-mediated cAMP response is abolished by sulpiride. Cultured striatal neurons (DIV 7) were incubated with VPA (1 mM) for 24 hrs followed by sulpiride (3 µM) and DA (10 µM) treatment. Sulpiride was pretreated for 30 min prior to DA treatment. cAMP levels from the cell lysates were measured using cAMP enzyme-immunoassays. Data are mean ± SEM values. (n = 4; *p<0.05, **p<0.01, ***p<0.001; One-way ANOVA with Bonferroni post hoc test).

Accumulating evidence shows that dopamine signaling may play important roles in the expression of genes that regulate normal mood [16]. Moreover, a recent study suggests the importance of the Par-4/DRD2 signaling pathway in major depression and bipolar disorder [18]. Thus, we aimed to verify the potential involvement of dopamine signaling in the mood stabilizing efficacy of VPA by examining whether chronic VPA treatment can affect dopamine-mediated cAMP signaling in cultured striatal neurons. Primary cultured mouse striatal neurons (DIV 7) were incubated with 1 mM VPA for 24 hrs then treated with an increasing concentration of dopamine for 30 min. Interestingly, VPA-treated neurons showed decreased cAMP levels compared to the control group in a concentration-dependent manner (Fig. 5A).

Because DRD2 activity has been implicated in normal mood control, we tested if the effect of VPA on dopamine signaling could be attributed to enhanced DRD2 activity using sulpiride, a specific DRD2 antagonist. Cultured striatal neurons (DIV 7) were incubated with 1 mM VPA for 24 hrs. The cells were then pretreated with 3 µM sulpiride for 30 min followed by 10 µM DA for 30 min. Sulpiride decreased the extent of cAMP reduction in VPA-treated neurons, indicating that DRD2 activity was enhanced by chronic VPA treatment (Fig. 5B). Taken together, these data show that chronic VPA treatment affects dopamine-mediated signaling in neurons, which is likely attributable to changes in DRD2 activity.

## Discussion

In this study, we provide evidence that chronic treatment of the common mood stabilizer, VPA, elicits robust induction of Par-4, which significantly impacts DRD2-mediated signaling. Furthermore, the induction mechanism involves dynamic chromatin remodeling in the Par-4 promoter regions and indicates that part of the mood stabilizing efficacy of VPA is mediated by changes in DRD2-mediated signaling processes.

Evidence for a link between the dopamine system and mood disorders has been consistently reported. For example, anhedonia and amotivation commonly seen in mood disorder patients can be reasonably explained by deficits in dopamine circuits [19]. In addition, the cerebrospinal fluid from depression patients who committed suicide often contains lower levels of dopamine metabolites [20]. When animals are exposed to chronic mild stress, a behavioral paradigm that induces a depressive state, a decrease in the surface expression of DRD2 in the nucleus accumbens occurs, which can be reversed by chronic administration of antidepressants [21]. Moreover, the immobility of experimental animals in Porsolt's forced swim test was reduced by D2-like dopamine receptor agonists [22]. Importantly, impaired DRD2-mediated signaling by functional defects in the Par-4 protein has been associated with depression-like behavior in animal models [16]. Consistent with this, one report showed that the Par-4/DRD2 signaling pathway in the striatum was modified by stress-induced depressive behavior through a non-methylation mechanism [23]. Moreover, a recent postmortem study showed that Par-4 protein levels were decreased by 31% and 67% in the temporal cortices of bipolar disorder and major depression patients, respectively, without significant decreases in the DRD2 levels [18]. Thus, our study not only further supports that the deregulation of dopamine signaling is involved in the pathogenesis of mood disorders, but also suggests that transcriptional upregulation may participate in the mechanism responsible for VPA-mediated mood stabilization.

The clinical effects of mood stabilizers and antidepressants are known to take a relatively long time to be achieved, which suggests the involvement of long lasting changes in the cellular environment. Chromatin remodeling has been proposed as a plausible mechanism to explain the delayed but enduring effect of mood stabilizers. In particular, VPA is known to harbor HDAC inhibitory activity that exerts an enduring impact on the chromatin state. In this study, we demonstrated that chronic VPA-associated alterations of DRD2 signaling may result from changes in chromatin states, which enhance the transcription of the Par-4 gene. It is especially noteworthy that Par-4 mRNA and protein accumulation did not increase until after several hours of treatment, which suggests a potential mechanism to explain the delayed clinical effect of VPA in human patients.

Lithium, another first line medication for bipolar disorder, has overlapping but distinct mechanisms with VPA as a mood stabilizer. For example, lithium mediates GSK-3β inhibition, which may be linked to neuroprotective effects against glutamate-induced toxicity [24]. Furthermore, the co-treatment of lithium and HDAC inhibitors shows a synergistic neuroprotective effect, suggesting the GSK-3β pathway as a common target for the therapeutic effect of lithium [24], [25], [26], [27], [28]. However, while lithium is known to directly inhibit GSK-3β, the effect of VPA on the GSK-3β pathway appears inconsistent depending on the experimental settings, hinting at the mechanistically distinct activities of VPA and lithium [29], [30], [31], [32], [33]. Interestingly, in our study, Par-4 protein levels were not effectively elevated by lithium. Thus, the result suggests that the modulation of the dopamine signaling pathway by Par-4 induction might be a unique process mediating the therapeutic efficacy of VPA, which is distinct from the mechanisms of action of other mood stabilizers.

## Materials and Methods

### Ethics Statement

This protocol was approved by the Pohang University of Science and Technology Institutional Animal Care and Use Committee (Approval ID: LIFE 020). This study was carried out in strict accordance with the recommendations in the Guide for the Care and Use of Laboratory Animals of the National Institutes of Health, and all efforts were made to minimize suffering.

### Primary culture of mouse neurons

Hippocampal and striatal regions were dissected from E14.5 embryos in Hank's balanced salt solution (HBSS, Invitrogen) without calcium or magnesium. The dissociated cells were diluted in plating media (Neurobasal for hippocampal neurons and MEM for striatal neurons (Invitrogen) supplemented with 1 M HEPES, pH 7.4, 10% glutamine, and 10% horse serum) and plated into 12-well or 24-well plates coated with poly-D-lysine with laminin. Two to four hours after the plating, the media were replaced with the appropriate culture media supplemented with 0.02% B27, 10% glutamine, and antibiotics.

### Drug treatment

Cultured neurons at DIV 7 were subjected to the drug treatment as indicated. VPA, sodium butyrate, trichostatin A, lithium chloride, carbamazepine, and lamotrigine were purchased from Sigma. Adult mice (C57BL/6, male, 9 weeks of age) were housed in a 12-h light/dark cycle, and intraperitoneally injected with saline or VPA (100 mg/kg) daily for 7 days. Mice were sacrificed at the indicated time points after the last injection of either VPA (100 mg/kg per day) or saline, and the brains were immediately dissected and processed for experimental procedures. For the cAMP assay, striatal neurons (DIV 7) were pretreated with 1 mM VPA for 24 hrs. After the VPA treatment, an increasing dose of dopamine (Sigma) was added for 30 min. Sulpiride (3 µM, Tocris) was pretreated for 30 min ahead of the dopamine treatment.

### cAMP enzyme-immunoassay

Media were replaced with fresh culture media (MEM supplemented with B27, glutamine and antibiotics) 1 day before drug treatment in striatal neurons cultured in 12-well plates. The cells were lysed in 400 µl of 0.1 M HCl solution for 20 min with gentle shaking and spun in microcentrifuge tubes. The cAMP content in the supernatant was measured using the cAMP-Enzyme Immunoassay Kit (Sapphire Bioscience).

### Chromatin immunoprecipitation (ChIP)

Hippocampal tissues and cultured primary neurons were lysed in the buffer containing 10 mM Tris-HCl, pH 8.1, 1% SDS, 0.5% Triton X-100, 10 mM EDTA, 0.5 mM EGTA and protease inhibitor cocktail (Roche). After sonication (amplitude 30, 15 sec, total 10 times, Vibra-Cells, Sonics & Materials, Inc), samples were subjected to ChIP assays immediately using the Upstate Biotechnology ChIP kit (Upstate Biotechnology) with slight modifications from previous reports [34], [35]. Anti-acetylated H3 K9, and K14 and anti-acetylated H4, K5, K8, K12, and K16 (#06-599 and #06-866, respectively; Millipore) antibodies were used for the ChIP assay. The immunoprecipitates were resuspended in H2O and used for semi-quantitative PCR analysis using the primer sets: Par-4 Forward: 5′-CGGTGTAGAGACAGCGTGAA-3′; Par-4 Reverse: 5′-GACTGAGAAACCCAGCAAGC-3′; GAPDH Forward: 5′-AACGACCCCTTCATTGAC-3′; GAPDH Reverse: 5′-TCCACGACATACGCAC-3′.

### RT-PCR and Quantitative real-time PCR

Total RNA was extracted using Tri-Solution (Bioscience Technology) from cultured neurons (DIV 7) or differentiated CAD cells. For reverse transcription, 1 µg RNA was used to synthesize first-strand cDNA using the SuperScript III system (Invitrogen). The PCR condition was one cycle of 95°C for 2 min; 21–32 cycles of 95°C for 1 min, 52°C or 56°C for 40 sec, and 72°C for 40 sec; and one cycle of 72°C for 7 min followed by 8°C.

For real-time PCR, 3 µL of 1/5-diluted cDNA and specific primers were mixed in a final volume of 20 µL in the SYBR premix Ex Taq (Takara). Quantitative real-time PCR was performed using the StepOnePlus real-time PCR system (Applied Biosystems). Primer sequences for PCR were;

mPar-4 Forward: 5′-AGGAAGCTGCGGGAGAAG-3′; mPar-4 Reverse: 5′-CAGGTAGGATGTGCCTGGAT-3′

HDAC1 Forward: 5′-TGCGTGGAAAGACAACC-3′; HDAC1 Reverse: 5′-ACCCCAGACCCCTCCTAAATG-3′

HDAC2 Forward: 5′-GGGACAGGCTTGGTTGTTTC-3′; HDAC2 Reverse: 5′-GAGCATCAGCAATGGCAAGT-3′

HDAC4 Forward: 5′-CAGGAGATGCTGGCCATGAA-3′; HDAC4 Reverse: 5′-GCACTCTCTTTGCCCTTCTC-3′

HDAC5 Forward: 5′-GCTTCTTTGGACCAGAGTTCC-3′; HDAC5 Reverse: 5′-CATCTCAGTGGGGATGTTGG-3′

GAPDH Forward: 5′-CACTGAAGGGCATCTTGG-3′; GAPDH Reverse: 5′-TTACTCCTTGGAGGCCATG-3′.

### Western blot analysis

Cultured neurons were lysed in 2X sampling buffer or 1X ELB lysis buffer (250 mM NaCl, 50 mM Tris-HCl, pH 8.0, 5 mM EDTA, 0.1% NP40, and a protease inhibitor cocktail). Protein concentrations were determined by the Bradford method, and 15–50 µg of protein was subjected to SDS-PAGE followed by Western blot analysis. The antibodies used were anti-Par-4 (sc-1807, 1∶2000, Santa Cruz Biotechnology), anti-α-Tubulin (sc-32293, 1∶2000, Santa Cruz Biotechnology), anti-GAPDH (sc-32233, 1∶2000, Santa Cruz Biotechnology), anti-acetylated H3 K9, and K14 (#06-599, 1∶2000, Millipore), anti-acetylated H4 K5, K8, K12 and K16 (#06-866, 1∶2000, Millipore), and anti-mouse and anti-rabbit HRP-labeled secondary antibodies (1∶15000; Thermo Scientific and KPL, respectively).

### Transfection

Central adrenergic tyrosine hydroxylase expressing (CATH) a-differentiated (CAD) cells were transfected using Lipofectamine 2000 (Invitrogen) followed by differentiation, as described previously [36], [37]. Briefly, the plasmids and Lipofectamine solution were mixed in Opti-MEM media (Invitrogen) and incubated for 15–20 min. The mixture was then incubated with cultured cells for 4–6 hrs. Before the transfection, media were replaced with serum-free DMEM media, and cells were cultured for an additional 5 days for differentiation.
