# Correction: Valproate Alters Dopamine Signaling in Association with Induction of Par-4 Protein Expression

**DOI:** 10.1371/annotation/49a3f8f0-b994-4d66-9f2e-65ef4929965b

**Published:** 2014-01-07

**Authors:** Saebom Lee, Jaehoon Jeong, Young-Un Park, Yongdo Kwak, Seol Ae Lee, Haeryun Lee, Hyeon Son, Sang Ki Park

Information regarding funding to the seventh author (HS) is incorrectly omitted from the Funding Statement. The following should be read with the Funding statement: 

“HS was supported by NRF Grant 2011-0028317 funded by the Ministry of Education, Science, and Technology, Republic of Korea.”

